# Severe Multivessel Coronary Vasospasm Secondary to 5-Fluorouracil Mimicking ST-Segment Elevation Myocardial Infarction

**DOI:** 10.1016/j.jscai.2023.101264

**Published:** 2023-12-23

**Authors:** Aryan Mehta, Mridul Bansal, Saraschandra Vallabhajosyula

**Affiliations:** a Department of Medicine, University of Connecticut School of Medicine, Farmington, Connecticut; b Department of Medicine, East Carolina University Brody School of Medicine, Greenville, North Carolina; c Division of Cardiology, Department of Medicine, Warren Alpert Medical School of Brown University, Providence, Rhode Island; d Lifespan Cardiovascular Institute, Providence, Rhode Island

**Keywords:** cardio-oncology, cardiotoxicity, coronary angiography, coronary vasospasm

**Figure 1:**
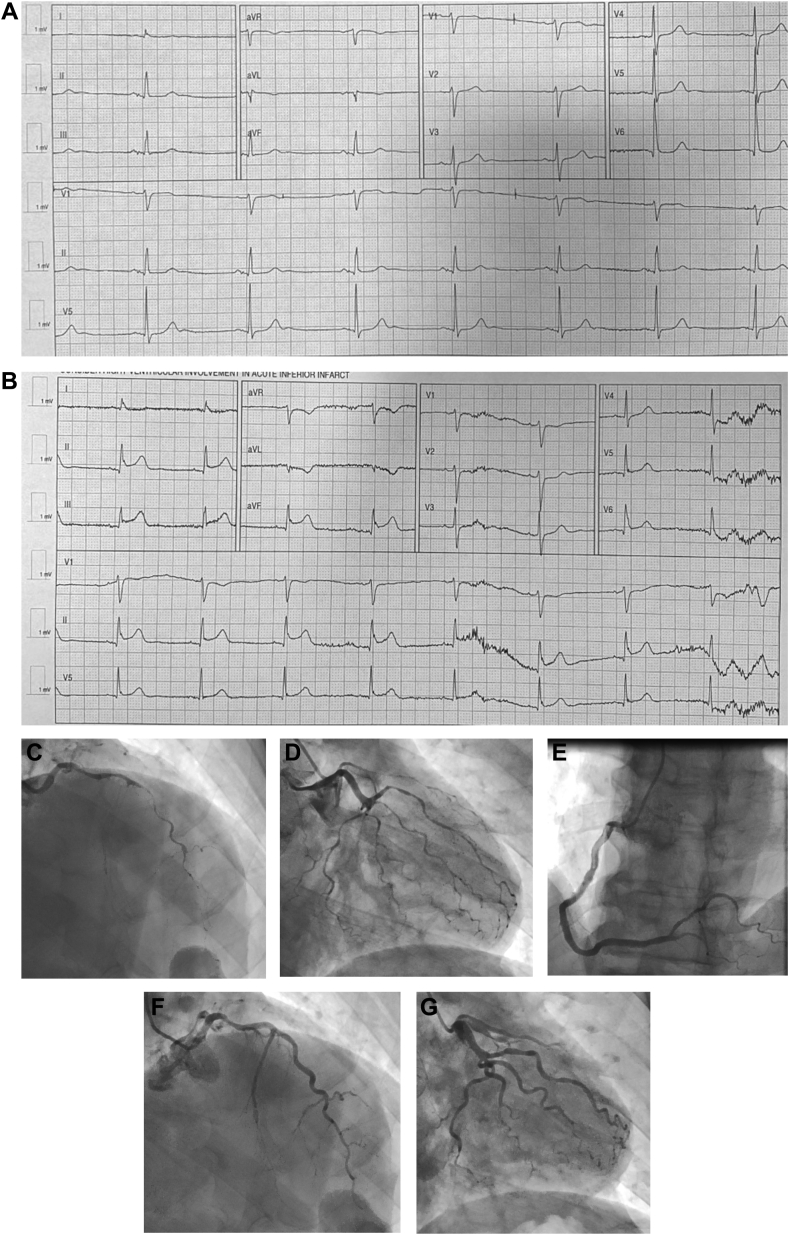
**ECG and coronary angiography in coronary vasospasm mimicking STEMI.** (**A**) Initial ECG demonstrating normal sinus rhythm with nonspecific ST-T–wave changes. (**B**) Subsequent ECG during chest pain “episode” demonstrate inferior STEMI. Coronary angiography demonstrating severe pruning of distal coronary vessels in the left anterior descending (**C**), left circumflex (**D**), and right coronary (**E**) arteries. Restoration of normal caliber of coronary arteries after injection of intracoronary nitroglycerin in the left anterior descending (**F**) and left circumflex (**G**) arteries. ECG, electrocardiogram; STEMI, ST segment–elevation myocardial infarction.

Five-Fluorouracil (5-FU) is frequently used in antineoplastic regimen, especially for colonic carcinoma. In rare cases, 5-FU may be associated with cardiotoxicity ranging from asymptomatic electrocardiographic changes to overt ischemia.^1^ A 56-year-old man with a history of hypertension, hyperlipidemia, cecal carcinoma on chemotherapy, and coronary artery disease (CAD) with prior right coronary artery stents presented with chest discomfort of 1-day duration. The chest discomfort worsened when he tried to stand. His home medications included atorvastatin, clopidogrel, carvedilol, nitroglycerine, and gabapentin. His chemotherapeutic regimen included leucovorin, 5-FU, and oxaliplatin of which he had completed 10 cycles. His initial electrocardiogram at an outside medical center was unremarkable (Figure 1A), but a subsequent electrocardiogram obtained during the chest pain episode demonstrated ST segment–elevation in the inferior leads (Figure 1B). His vital signs were stable and laboratory parameters unremarkable. Initial high-sensitivity troponin was elevated at 145 ng/L, and bedside transthoracic echocardiography noted a left ventricular ejection fraction of 45% to 50% with anterolateral and inferolateral hypokinesis. Emergent coronary angiography demonstrated multivessel CAD involving left anterior descending, left circumflex, and distal right coronary arteries concerning for coronary vasospasm versus spontaneous coronary artery dissection (Figure 1C-E and Supplemental Videos 1-3). This resolved with the instillation of intracoronary nitroglycerin, suggestive of coronary vasospasm as the etiology (Figure 1F-G and Supplemental Videos 4 and 5). The coronary angiogram after instillation of intracoronary nitroglycerin showed mild nonobstructive CAD without significant occlusive stenosis. The patient was started on oral long-acting nitroglycerin and calcium channel blockers due to presumed coronary artery vasospasm secondary to 5-FU use. His 5-FU therapy was discontinued as an outpatient, and alternate methods were used to manage his cecal carcinoma. He was asymptomatic without recurrences at 6-month follow-up.

Cardiotoxicity from 5-FU is an infrequent side effect seen in <20% of the population presenting as angina, palpitations, and dyspnea.^1^ Less commonly, it can present as an acute myocardial infarction, arrhythmia, cardiac arrest, and heart failure.^2^ 5-FU and its active metabolites result in endothelial dysfunction.^3^ Intrinsically abnormal coronary vasculature, with mild atherosclerosis, have a higher predilection for spasm due to abnormal endothelial function.^4^ Studies have elucidated the role of endothelin-1, endothelial nitric oxide synthases, protein kinase C, and acetylcholine, which might alter local coronary vasculature and cause coronary vasoconstriction.^3^ Increased release of endothelin-1 (vasoconstrictor) and decreased release of prostacyclin (vasodilator) from a diseased endothelium result in vasospasm in these patients.^3^^,^^4^ A single-center study spanning over almost a decade reported the incidence of coronary vasospasm after being treated with 5-FU to be ∼2%.^5^ Management often involves cessation of 5-FU if it is suspected to be the causative agent along with empiric treatment with coronary vasodilators (long-acting nitrates and calcium channel blockers).^3^ Often there might be complete cessation of vasospasm after withdrawal of the causative agent without the need of additional therapy. Given the lack of alternatives, a rechallenge of 5-FU might be needed. However, the risk of recurrence of cardiotoxicity is as high as ∼50% in some cases.^2^ Prophylactic treatment with nitrates and calcium channels blockers have been explored but with varying success rates.
